# Continuous Flow Separation of Live and Dead Cells Using Gravity Sedimentation

**DOI:** 10.3390/mi14081570

**Published:** 2023-08-08

**Authors:** Adem Ozcelik, Sinan Gucluer, Tugce Keskin

**Affiliations:** Department of Mechanical Engineering, Aydin Adnan Menderes University, Aydin 09010, Türkiye; sgucluer@adu.edu.tr (S.G.);

**Keywords:** microfluidic cell separation, microfluidics, continuous flow cell separation, lab-on-chip

## Abstract

The separation of target cell species is an important step for various biomedical applications ranging from single cell studies to drug testing and cell-based therapies. The purity of cell solutions is critical for therapeutic application. For example, dead cells and debris can negatively affect the efficacy of cell-based therapies. This study presents a cost-effective method for the continuous separation of live and dead cells using a 3D resin-printed microfluidic device. Saccharomyces cerevisiae yeast cells are used for cell separation experiments. Both numerical and experimental studies are presented to show the effectiveness of the presented device for the isolation of dead cells from cell solutions. The experimental results show that the 3D-printed microfluidic device successfully separates live and dead cells based on density differences. Separation efficiencies of over 95% are achieved at optimum flow rates, resulting in purer cell populations in the outlets. This study highlights the simplicity, cost-effectiveness, and potential applications of the 3D-printed microfluidic device for cell separation. The implementation of 3D printing technology in microfluidics holds promise for advancing the field and enabling the production of customized devices for biomedical applications.

## 1. Introduction

The purity of cell solutions is critical in various fields of biological research and medical applications [1]. Maintaining a high level of purity ensures accurate experimental results and enhances the reliability of downstream analyses [2,3]. Pure cell populations are crucial for studying cell behavior, signaling pathways, and disease mechanisms, as well as for developing targeted therapeutics [4,5]. Especially for cell-based therapies, removing dead cells and debris from cell solutions is critical for the treatment efficacy [6,7].

In several branches of biology, bioengineering, and healthcare, sorting and separating cells from complicated, heterogeneous mixtures constitutes a crucial challenge [8,9]. To boost productivity in research and development applications, cell sorting is frequently employed to concentrate or purify cell specimens into clearly defined populations [10]. In 1969, Herzenberg et al. developed the earliest commercialized cell sorter, which uses a method generally referred to as FACS [11]. In conventional FACS, a focused laser beam is diffused over a detector when it comes into contact with fluorescently labeled cells organized in laminar flow. FACS systems can be implemented to separate viable and nonviable cells through compatible cell-staining. However, commercial FACS systems are fairly expensive tools that can be frequently found in well-established centralized laboratories [12,13]. For researchers around the world, simpler and more affordable methods are required to enable separation of dead and live cells. The field of microfluidics offers alternative approaches to cell manipulation and separation by implementing miniaturized fluidic channels and actuation mechanisms [13,14,15,16,17]. Including cell separation, microfluidic platforms have been employed in a wide range of applications in biomedical research [18,19,20,21].

Microfluidic cell separation can be generally categorized into labeled and label-free methods. Labeled microfluidic cell sorting methods harness the power of fluorescent or magnetic labels that are attached to specific cell markers or antigens [22,23]. These labels can be introduced using fluorescent dyes or magnetic nanoparticles conjugated to antibodies. During the sorting process, the labeled cells flow through microfluidic channels, where they encounter optical or magnetic forces [24]. These forces enable the separation of target cells based on their unique markers or antigens. Various mechanisms, such as hydrodynamic forces, dielectrophoresis, or magnetic field gradients, are then employed to selectively divert or collect the labeled cells [25,26]. These techniques offer exceptional specificity and sensitivity, enabling researchers to precisely isolate target cells from complex mixtures. In contrast, label-free microfluidic cell sorting methods take a different approach by not relying on external labels or markers to differentiate cells [27,28,29]. Instead, they capitalize on the inherent physical or biochemical properties of cells, including size, shape, deformability, electrical impedance, or refractive index [30,31]. By exploiting these distinctive characteristics, label-free sorting methods enable the sorting of cells based on the variations in these properties. Label-free microfluidic cell sorting techniques offer notable advantages such as speed and reduced sample preparation steps, making them highly desirable for a wide range of applications [32].

Various microfluidic platforms and separation methods have been applied for separating live and dead cells [33,34,35]. For example, a recent study presented a microfluidic device that can sort live and dead cells based on differences in cell stiffness [36]. The device uses periodic ridges to compress cells and measure their deformability, allowing for the separation of live and dead cells with high accuracy. The study found that the sorted cells could be further processed after sorting and cultured for up to seven days. Zalis et al. demonstrated the use of acoustophoresis, a gentle method for the concentration of live and viable cells with high purity, without the need for protein fluorescent labeling [36]. The study examines the effectiveness of this method on different cell populations, including N2a cells and primary neurons. The study also discusses the clinical and pre-clinical applications of this method, as well as its advantages over other methods of cell concentration. Similarly, Olofsson et al. implemented acoustophoresis for effectively separating viable and dead cells without relying on cell size [33]. The researchers manipulated the acousto-mechanical properties of cells by employing a high-density medium. Through this innovative approach, they successfully demonstrated the ability to differentiate viable and dead cells by analyzing their distinct acoustic scattering properties. Yildizhan et al. showed the dielectrophoretic separation and characterization of live and dead monocytes using 3D carbon electrodes [34]. The method effectively removes dead cells while preserving the viability of live monocytes. By enriching the population of live monocytes, this approach reduces the risk of dead-cell contamination and enhances the reliability and accuracy of blood analyses and disease diagnosis.

Gravity-based sedimentation of different density particles and cells have been demonstrated previously using various designs of conventional microfluidic devices [28,37,38,39]. For example, one study investigates the use of the gravitational split-flow thin channel (G-SPLITT) system as a cell sorter for biomaterial fractionation [39]. The system separates myeloma cells from healthy splenocytes based on their different sedimentation rates. Huh et al. presents a microfluidic sorting system that achieves rapid (<1 min) and highly pure (>99.9%) separation of particles [38]. The system utilizes gravity and hydrodynamic flows to amplify sedimentation-based separation between particles, enabling efficient size profiling and continuous mass-dependent separation. Norouzi et al. demonstrated a microfluidic chip capable of sorting cells based on their density [37]. The chip utilizes a horizontal flowing micron-scale density gradient, where cells move vertically until their density matches the surrounding fluid. The chip successfully sorted polymer microbeads and blood cells, demonstrating high enrichment rates. Even though gravitational sedimentation has been employed for cell and particles, fabrication of these devices is not simple and requires specialized equipment. Furthermore, the separation of live and dead cells through continuous sedimentation has not been demonstrated before.

In general, existing microfluidic cell separation applications demonstrate the feasibility of a miniaturized system to isolate dead cells from cell solutions, but they generally rely on complicated external setups or require complex and expensive device fabrication steps [40,41,42]. In this study, we demonstrate a simple 3D resin-printed microfluidic device to continuously sperate live and dead yeast cells based on density differences using gravity sedimentation. Continuous separation of live and dead cells by differential sedimentation within a 3D-printed microfluidic device is shown for the first time.

## 2. Materials and Methods

**Figure 1:**
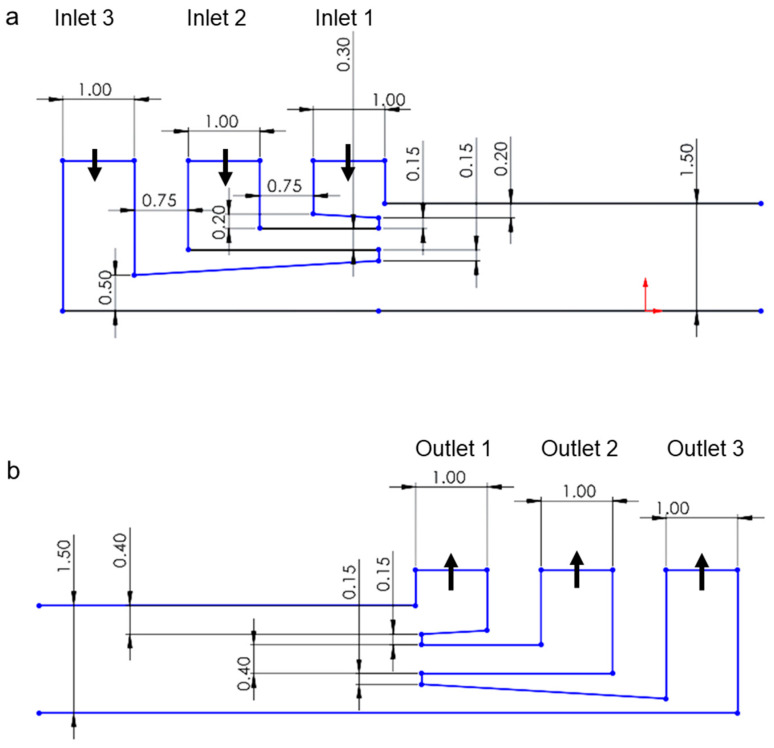
Detailed dimensions of (**a**) inlets and (**b**) outlets of the device. All dimensions are in millimeters. Arrows indicate the flow direction.

**Figure 2:**
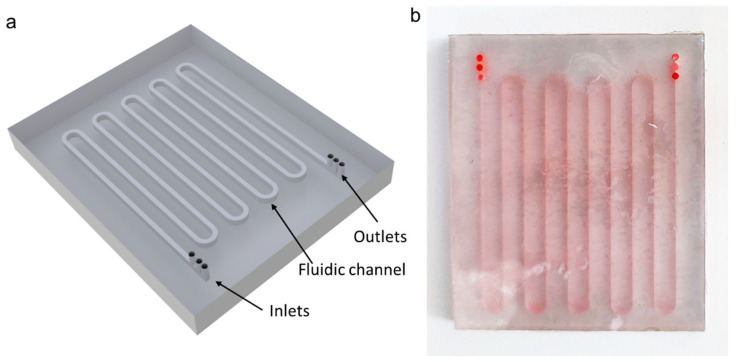
(**a**) Schematic depiction of the device. (**b**) Actual picture of the 3D-printed device.

A fluidic channel was designed with three inlets and three outlets. A three-inlet design was chosen to provide a well-focused stream of yeast cells so that cell sedimentation can be more uniform starting from approximately similar heights. At different initial flow rates, the sedimentation amount of dead and live cells can change significantly. Therefore, a three-outlet design was applied to analyze cell population purities at different heights of the device outlet. The specific dimensions and geometry of the inlets and outlets can be observed in Figure 1. The dimensions of the three inlets were chosen to minimize the top flow so that yeast cells could be focused and be as close as possible to the top of the channel ceiling. This way, cell sedimentation could be sufficient for live and dead cell separation. Outlets were divided into equal widths of 400 µm with 150 µm separating walls. The fluidic channel was designed with a total length of 400 mm, a width of 1 mm, and a height of 1.5 mm. Figure 2 showcases both a 3D schematic model and the physical device that was printed. The device was intentionally designed with a serpentine pattern to minimize its overall size while still achieving a channel length of at least 400 mm. Since very small flow rates were used in this study compared to inertial cell separation studies in the literature [43,44], the effect of these curvatures to the cell sedimentation is negligible. For the fabrication of the device, a commercial resin printer (Photon S, Anycubic, Shenzhen, Guangdong, China) was implemented. The 3D printer employs a 2560 × 1440 pixel UV LCD screen to create detailed prints with fine resolution. The printer uses a 405 nm UV LED light source to cure the resin with a build volume of 115 mm *×* 65 mm *×* 165 mm. A clear resin (Anycubic, Shenzhen, Guangdong, China) was used to print the device. For printing parameters, 25 micrometer layer height, 60 s initial exposure, and 7 s exposure at each layer were used.

For the cell separation experiments, *Saccharomyces cerevisiae* yeast cells were grown in Sabouraud dextrose (Sigma Aldrich, St. Louis, MO, USA) broth within a shake incubator operating at 30 °C. Following an incubation period of approximately 24 h, the culture underwent centrifugation at 10,000× *g* for 10 min to concentrate the yeast cells, and the resulting supernatant was carefully discarded. To suspend the cells, 2 mL of a 0.85% NaCl solution was added. Next, 1 mL of this cell suspension was separately introduced to two 30–40 mL centrifuge tubes that initially contained 20 mL of 0.85% NaCl for live yeast and 70% isopropyl alcohol for dead yeast cells. Both samples, comprising live and dead yeast, were incubated at room temperature for 1 h, with intermittent stirring every 10 min. After the incubation, both sets of cells were subjected to centrifugation at 10,000× *g* for 10 min, followed by re-suspension in separate tubes containing 10 mL of 0.85% NaCl. Prior to the experimental phase, both live and dead yeast cells were rinsed at least three times with deionized water utilizing a mini centrifuge (Cf8, Labtron, Surrey, UK). The live and dead yeast cells were subsequently re-suspended in a 1 mM phosphate buffer solution (PBS) (Sigma Aldrich, St. Louis, MO, USA), resulting in a final concentration of 10^6^ cells per mL. A 1% methylene blue solution is used for staining to observe the dead cells. After the staining, dead cells were washed 3 times and resuspended in PBS solution. At the final cell solutions, the ratio of the live and dead cells was 1:1. For the experiments, a three-channel custom-built syringe pump is used [45]. The inlets and outlets of the fluidic device were connected via silicone tubes with 1 mm outer diameter and 0.5 mm inner diameter. A standard hemocytometer was used to characterize live and dead cells under an inverted microscope (SOIF BK500, Guangzhou, China). The ratios of live and dead cells for each outlet were calculated for different sample flow rates.

The behavior of particles within fluidic channels is investigated through the implementation of the finite element method using COMSOL Multiphysics. For this, drag force and gravitational force were implemented, and particle tracing was performed to analyze the path of particles as a function of their densities. The drag force can be given as the following:

(1) $$F_{D}=\frac{1}{\tau_{p}}m_{p}\left(u-v\right)$$

(2) $$\tau_{p}=\frac{\rho_{p}d_{p}^{2}}{18\mu }$$

where *m_p_*, *u*, *v*, ρ*_p_*, *d_p_*, and *µ* are mass of the particle, fluid speed, particle speed, particle density, particle diameter, and viscosity of the fluid, respectively. The gravitational force can be defined as:

(3) $$F_{g}=m_{p}g\frac{\rho_{p}-\rho }{\rho_{p}}$$

where ρ is the fluid density and *g* is the gravitational acceleration. The motion of a particle can be described as:

(4) $$\frac{d}{dt}\left(m_{p}v\right)=F_{D}+F_{g}+F_{ext}$$

where *F_D_*, *F_g_*, and *F_ext_* are drag force, gravitational force and any other external force applied to the particle, respectively.

## 3. Results

### 3.1. Numerical Simulation

**Figure 3:**
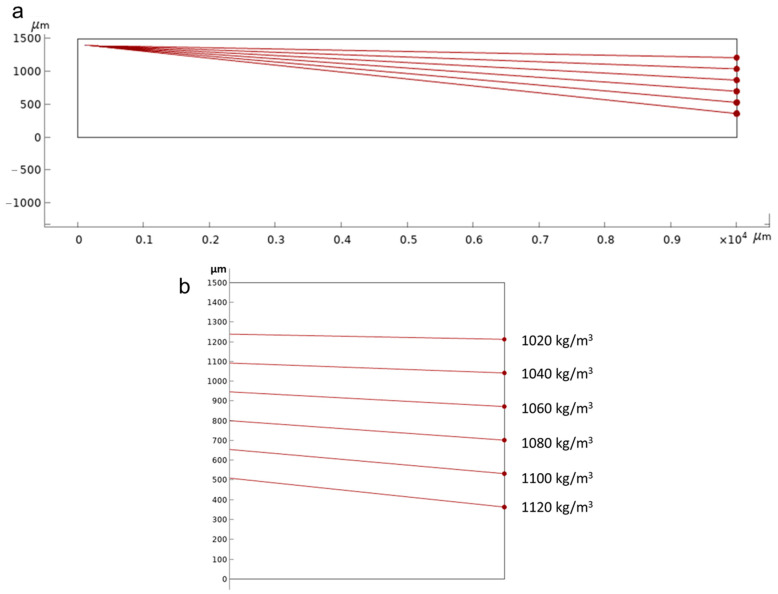
(**a**) Particle trajectories were simulated for particles with densities ranging from 1020 kg/m^3^ to 1120 kg/m^3^. (**b**) Zoomed image of the exit of the channel.

**Figure 4:**
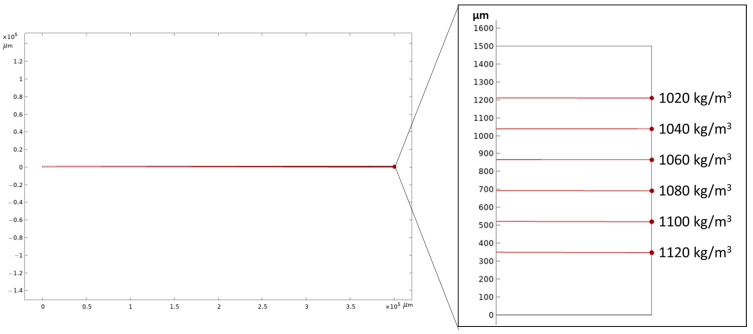
The sedimentation of particles based on density was simulated within a 400-mm-long fluidic channel for the flow velocity of 0.0004 m/s corresponding to a flow rate of 36 µL/min for the channel cross section of 1.5 mm *×* 1 mm. A zoomed-in view of the channel’s end is presented in the right panel.

To facilitate the visualization of particle trajectories throughout the entire channel, the fluidic channel is initially modeled as a rectangular shape measuring 1.5 mm *×* 10 mm. For shorter fluidic channels, an initial particle velocity of 0.00001 m/s is selected to observe significant particle sedimentation. This velocity corresponds to a flow rate of 0.9 µL/min for a channel cross-section of 1.5 mm *×* 1 mm. Numerical simulations are conducted in a two-dimensional format, representing cross-sections of the fluidic channel. Figure 3a illustrates the plotting of particle trajectories when considering gravitational force, buoyancy and drag force for spherical particles with a diameter of 5 μm, closely resembling yeast cells. The fluid density in the plot is set at 1000 kg/m^3^, while particle densities ranging from 1020 to 1120 kg/m^3^ are selected, encompassing the density range reported for yeast cells in existing literature [46]. To provide a clear observation, Figure 3b presents a magnified view of the channel’s end, featuring labeled particle density values. Additionally, a scaled-up fluidic channel with a length of 400 mm is simulated to observe the particle distribution with increasing particle density, depicted in Figure 4.

**Figure 5:**
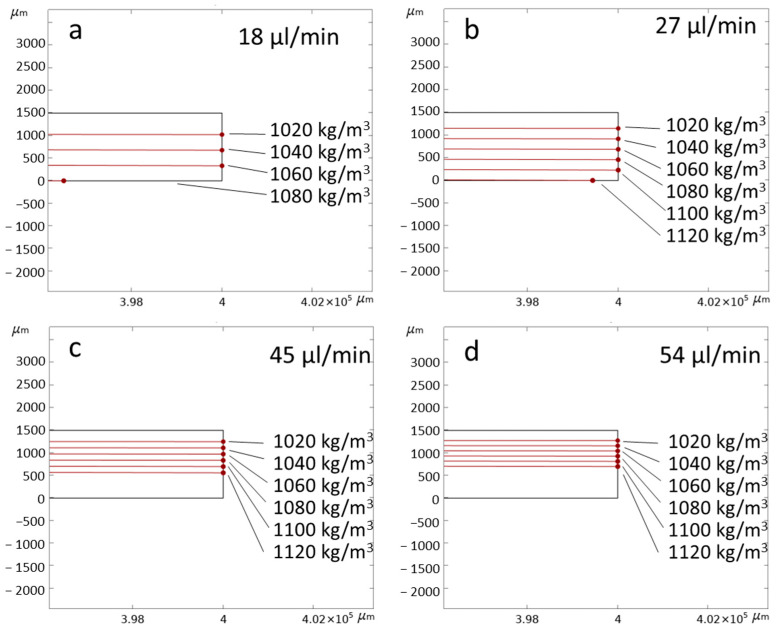
At the end of the 400-mm-long fluidic channel, particle trajectories were observed for different flow rates: (**a**) 18 µL/min, (**b**) 27 µL/min, (**c**) 45 µL/min, and (**d**) 54 µL/min.

In Figure 4, the initial fluid velocity was set to 0.0004 m/s, corresponding to a flow rate of 36 µL/min for the channel’s cross-section. To investigate particle behavior under varying flow rates, particle trajectories were simulated at the end of a 400 mm fluidic channel for increasing flow rates, as illustrated in Figure 5. The particle densities in the figures are consistent with those presented in Figure 4. In Figure 5a,b, only the first three particle densities reached the end of the channel. Particles at zero height mean that these particles were sedimented completely at these points and are shown as stuck to the bottom wall. However, in Figure 5c,d, as well as Figure 4 (where the flow rate is 36 µL/min), all particles within the given density range successfully reached the end of the fluidic channel.

### 3.2. Experimental Characterization of Sedimentation

To align with the numerical simulations, the experimental studies involved selecting five distinct total flow rates: 18 µL/min, 27 µL/min, 36 µL/min, 45 µL/min, and 54 µL/min, each representing a different fluid velocity. The device was designed with three inlets to ensure a well-focused stream of yeast cells and minimize wall effects during the experiments. To evaluate the efficiency of separating dead and live yeast cells, the ratio of live to dead cell counts was determined for samples collected from each outlet of the device. This involved first determining the total yeast cell numbers for each outlet solution using standard hemocytometer counting. Next, the number of stained cells was obtained, allowing for the calculation of the ratio between dead and live cells. This method was chosen to ensure the purity of each outlet, specifically containing only live cells, as the aim of this work is to isolate dead cells from the cell solution and provide higher-purity viable cell samples.

**Figure 6:**
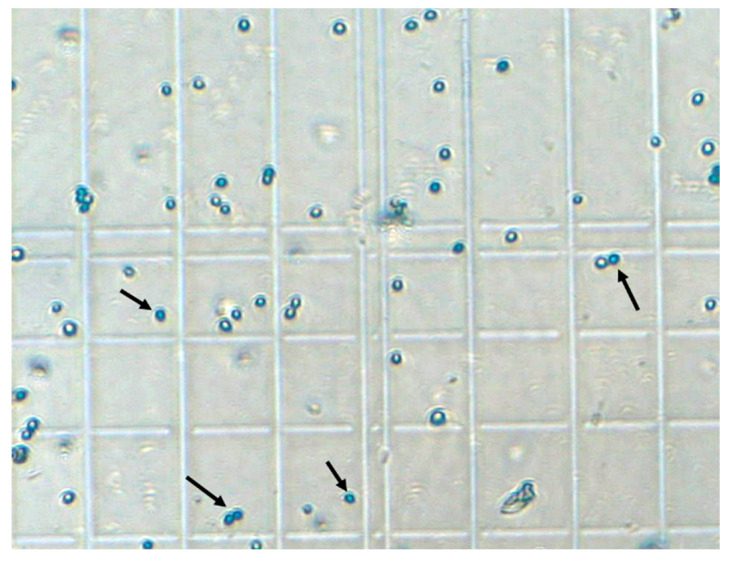
Example image of the collected yeast cells. Arrows show some of the stained cells.

**Figure 7:**
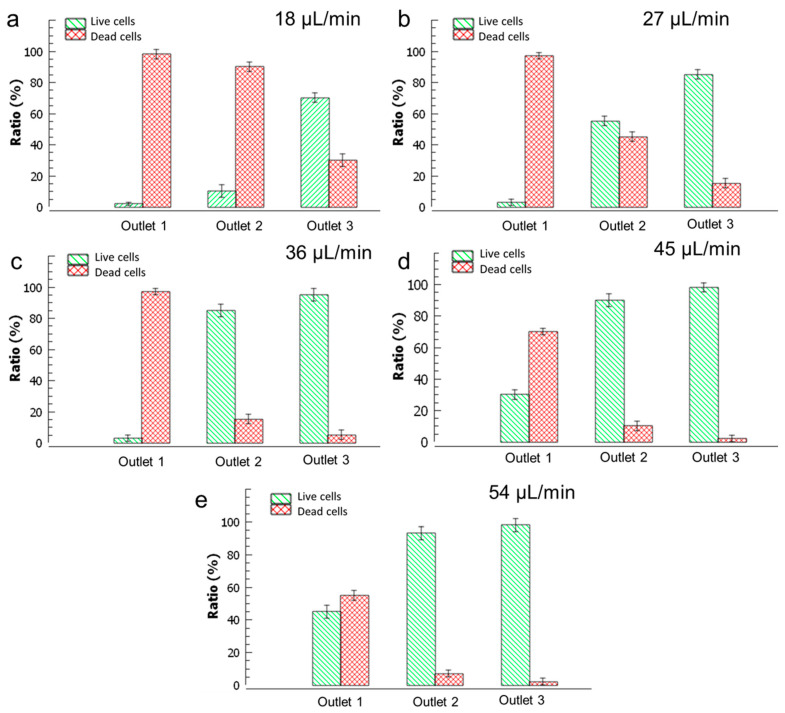
Calculated live and dead yeast cell ratios for the total flow rates of (**a**) 18 µL/min (**b**) 27 µL/min, (**c**) 36 µL/min, (**d**) 45 µL/min, and (**e**) 54 µL/min. Error bars represent standard error for three measurements.

For the 18 µL/min total flow rate, the yeast cell solution flow rate was set to 3 µL/min, while the upper and lower inlets were set to 3 µL/min and 12 µL/min, respectively. All the experimental flow conditions for the five total flow rates were given in Table 1. An example image of the collected yeast cells on the counting grids is shown in Figure 6. Figure 7a presents the ratios of live and dead yeast cells for the three outlets, where the red bars represent the dead cells, and the green bars represent the live (viable) cells. Similar plots characterizing the cells for total flow rates of 27 µL/min, 36 µL/min, 45 µL/min, and 54 µL/min are given in Figure 7b, Figure 7c, Figure 7d, and Figure 7e, respectively.

## 4. Discussion

Cell-based therapies play a crucial role in the research and treatment of various human diseases. These therapies, also known as cellular therapies or regenerative medicine, involve the use of living cells to prevent or treat diseases. They have the potential to transform disease treatment by regenerating damaged tissue, stimulating the body’s own repair mechanisms, and modifying immune responses. Stem cell therapy, gene therapy, and immune cell therapy are some examples of cell-based therapies. Stem cell therapy utilizes undifferentiated cells, known as stem cells, which can develop into different cell types in the body. These stem cells can be sourced from bone marrow, fat tissue, or umbilical cord blood. They can be employed to repair damaged tissue, such as through bone marrow transplants for leukemia treatment, or to stimulate natural healing processes, such as using stem cell injections to address osteoarthritis. However, for cell-based therapies to be effective and safe, it is crucial to separate dead cells from the cell population. By doing so, the efficiency of the therapies can be improved, while potential toxic effects can be minimized. Therefore, the development of practical methods for separating dead cells from cell solutions holds significant value.

In this study, we explore the potential of gravity-based sedimentation for continuously separating dead and live cells. Initially, numerical simulations were conducted. These simulations involved gradually increasing the density values of particles with a diameter of 5 μm, representing yeast cells. The selected density values ranged from the density of water at room temperature to the density of yeast cells, approximately 1095 ± 11 kg/m^3^. Figure 3 and Figure 4 illustrate the sedimentation and movement of particles towards the end of the channel based on their density. As expected, particles with sediment densities lower than those with higher densities demonstrated the possibility of separating dead and live cells based on their density differences.

Figure 4 presents simulation conducted in a 400-mm-long fluidic channel, demonstrating reasonable separation differences between particles of varying densities at acceptable total flow rates for potential application in actual experiments. The simulations encompassed total flow rates of 18 µL/min, 27 µL/min, 36 µL/min, 45 µL/min, and 54 µL/min, resulting in distinct particle trajectories, as illustrated in Figure 5. The desired outcome is well-separated particles reaching the end of the fluidic channel. This separation is observed at a total flow rate of 36 µL/min, where particles of different densities are suitably spaced and reach the channel’s end. Higher flow rates tend to reduce particle separation, while lower flow rates cause lower-density particles to sink further and contaminate lower collection outlets.

When a cell’s membrane becomes damaged or the cell dies, its contents can be exposed to the surrounding environment. This exposure may occur due to physical damage, disease, injury-induced cell death, or the action of specific enzymes that can degrade the cell membrane. If the cell is in a liquid medium, such as a cell culture or bodily tissues, the medium can enter the cell through the damaged membrane. Consequently, it is expected that the cell density would be influenced by the density of the surrounding medium. In this study, water is used as the cell medium, with a density of approximately 1000 kg/m^3^ at room temperature. Therefore, dead yeast cells are anticipated to have lower densities compared to live cells (density range of 1095 ± 11 kg/m^3^).

In the experimental setup, the mixture of dead and live yeast cells was passed through the fluidic device for each total flow rate, and the liquid samples collected from each outlet were analyzed to determine cell ratios. At the lowest total flow rate (18 µL/min), the first and second outlets predominantly contained over 90% dead cells, while the third outlet had approximately 30% dead cells, as displayed in Figure 7a. This indicates poor separation efficiency at this flow rate, with the live cells highly contaminated by dead cells. Similar observations can be made from Figure 7b, where liquid samples collected from the second and third outlets exhibit high contamination with dead cells. At a total flow rate of 36 µL/min, the samples collected from all three outlets demonstrate improved purity and reduced contamination, as shown in Figure 7c. The first mainly contains dead cells (∼97%), the second outlet mainly contains of live cells (∼85%), and the third outlet mainly contains live cells (∼95%). As the total flow rate increases further, the presence of live cells in the first outlet leads to the contamination of live cells with dead cells and decreased separation efficiency, as depicted in Figure 7d,e. In summary, Figure 7 shows that at lower than 36 µL/min total flow rates, dead cells will have enough time to sediment as much as live cells and contaminate the outlets at lower heights. Similarly, at total flow rates above 36 µL/min, live cells that have larger densities will not have enough time to sediment to the heights to enter the lower outlets.

Overall, these results demonstrate the potential benefit of a simple device for continuous separation of live and dead cells based on density differences. Compared to the existing cell separation methods, the presented approach is simple and low-cost and is critical for many researchers in the low-resourced settings. Considering the benefits of the 3D-printed device for cell purification, it can be potentially implemented in many lab-on-chip applications within the fields of biology and medicine.

## 5. Conclusions

In this study, we present a cost-effective and user-friendly method for cell separation using standard resin 3D printing technology. We utilized numerical simulations to investigate the impact of different flow rates on the sedimentation of particles with varying densities. Building upon the numerical findings, we performed experiments and analyzed the collected dead and live yeast cells from different outlets of our device. The results highlight the feasibility of employing low-cost microfluidic devices fabricated through 3D printing in cell separation experiments. The utilization of 3D printing has the potential to revolutionize the field of microfluidics by enabling the efficient and economical production of microfluidic devices. Traditional fabrication techniques such as photolithography, injection molding, and laser ablation can be expensive, time-consuming, and less suitable for producing intricate or small-scale microfluidic structures.

## Figures and Tables

**Table 1:** Experimental flow conditions for the three inlets.

Inlet 1	Inlet 2 (Sample)	Inlet 3	Total Flow Rate
3 µL/min	3 µL/min	12 µL/min	18 µL/min
4.5 µL/min	4.5 µL/min	18 µL/min	27 µL/min
6 µL/min	6 µL/min	24 µL/min	36 µL/min
7.5 µL/min	7.5 µL/min	30 µL/min	45 µL/min
9 µL/min	9 µL/min	36 µL/min	54 µL/min

## Data Availability

Data is available upon reasonable request from the corresponding author.

## References

[1] Bao Q., Zhao Y., Niess H., Conrad C., Schwarz B., Jauch K.-W., Huss R., Nelson P.J., Bruns C.J. (2012). Mesenchymal Stem Cell-Based Tumor-Targeted Gene Therapy in Gastrointestinal Cancer. Stem Cells Dev..

[2] Chiu T.-K., Chou W.-P., Huang S.-B., Wang H.-M., Lin Y.-C., Hsieh C.-H., Wu M.-H. (2016). Application of Optically-Induced-Dielectrophoresis in Microfluidic System for Purification of Circulating Tumour Cells for Gene Expression Analysis-Cancer Cell Line Model. Sci. Rep..

[3] Cheng J., Liu Y., Zhao Y., Zhang L., Zhang L., Mao H., Huang C. (2020). Nanotechnology-Assisted Isolation and Analysis of Circulating Tumor Cells on Microfluidic Devices. Micromachines.

[4] Phillips M.A., Gran M.L., Peppas N. (2010). a Targeted Nanodelivery of Drugs and Diagnostics. Nano Today.

[5] Meng L., Cai F., Jiang P., Deng Z., Li F., Niu L., Chen Y., Wu J., Zheng H. (2014). On-Chip Targeted Single Cell Sonoporation with Microbubble Destruction Excited by Surface Acoustic Waves. Appl. Phys. Lett..

[6] Chan J.K.Y., Lam P.Y.P. (2013). Human Mesenchymal Stem Cells and Their Paracrine Factors for the Treatment of Brain Tumors. Cancer Gene Ther..

[7] Li Z., Xu H., Yu L., Wang J., Meng Q., Mei H., Cai Z., Chen W., Huang W. (2022). Patient-derived Renal Cell Carcinoma Organoids for Personalized Cancer Therapy. Clin. Transl. Med..

[8] Wu M., Ozcelik A., Rufo J., Wang Z., Fang R., Jun Huang T. (2019). Acoustofluidic Separation of Cells and Particles. Microsyst. Nanoeng..

[9] Ozcelik A., Cevik O. (2023). Microfluidic Methods Used in Exosome Isolation. Biocell.

[10] Gao Y., Wu M., Lin Y., Xu J. (2020). Acoustic Microfluidic Separation Techniques and Bioapplications: A Review. Micromachines.

[11] Herzenberg L.A., Parks D., Sahaf B., Perez O., Roederer M., Herzenberg L.A. (2002). The History and Future of the Fluorescence Activated Cell Sorter and Flow Cytometry: A View from Stanford. Clin. Chem..

[12] Nawaz A.A., Chen Y., Nama N., Nissly R.H.R.H., Ren L., Ozcelik A., Wang L., McCoy J.P.P., Levine S.J.S.J., Huang T.J.T.J. (2015). Acoustofluidic Fluorescence Activated Cell Sorter. Anal. Chem..

[13] Fu A.Y., Spence C., Scherer A., Arnold F.H., Quake S.R. (1999). A Microfabricated Fluorescence-Activated Cell Sorter. Nat. Biotechnol..

[14] Johansson L., Nikolajeff F., Johansson S., Thorslund S. (2009). On-Chip Fluorescence-Activated Cell Sorting by an Integrated Miniaturized Ultrasonic Transducer. Anal. Chem..

[15] Lin Y., Gao C., Gao Y., Wu M., Ahmadian Yazdi A., Xu J. (2019). Acoustofluidic Micromixer on Lab-on-a-Foil Devices. Sens. Actuators B Chem..

[16] Wu M., Gao Y., Ghaznavi A., Zhao W., Xu J. (2022). AC Electroosmosis Micromixing on a Lab-on-a-Foil Electric Microfluidic Device. Sens. Actuators B Chem..

[17] Gao Y., Wu M., Lin Y., Zhao W., Xu J. (2020). Acoustic Bubble-Based Bidirectional Micropump. Microfluid. Nanofluidics.

[18] Cai H., Ao Z., Wu Z., Song S., Mackie K., Guo F. (2021). Intelligent Acoustofluidics Enabled Mini-Bioreactors for Human Brain Organoids. Lab Chip.

[19] Ao Z., Cai H., Wu Z., Ott J., Wang H., Mackie K., Guo F. (2021). Controllable Fusion of Human Brain Organoids Using Acoustofluidics. Lab Chip.

[20] Ao Z., Cai H., Wu Z., Krzesniak J., Tian C., Lai Y.Y., Mackie K., Guo F. (2022). Human Spinal Organoid-on-a-Chip to Model Nociceptive Circuitry for Pain Therapeutics Discovery. Anal. Chem..

[21] Wu Y., Ao Z., Chen B., Muhsen M., Bondesson M., Lu X., Guo F. (2018). Acoustic Assembly of Cell Spheroids in Disposable Capillaries. Nanotechnology.

[22] Ren L., Yang S., Zhang P., Qu Z., Mao Z., Huang P.-H., Chen Y., Wu M., Wang L., Li P. (2018). Standing Surface Acoustic Wave (SSAW)-Based Fluorescence-Activated Cell Sorter. Small.

[23] Modak N., Datta A., Ganguly R. (2009). Cell Separation in a Microfluidic Channel Using Magnetic Microspheres. Microfluid. Nanofluidics.

[24] Hejazian M., Li W., Nguyen N.-T. (2015). Lab on a Chip for Continuous-Flow Magnetic Cell Separation. Lab Chip.

[25] Witek M.A., Freed I.M., Soper S.A. (2020). Cell Separations and Sorting. Anal. Chem..

[26] Bhagat A.A.S., Bow H., Hou H.W., Tan S.J., Han J., Lim C.T. (2010). Microfluidics for Cell Separation. Med. Biol. Eng. Comput..

[27] Menachery A., Kumawat N., Qasaimeh M. (2017). Label-Free Microfluidic Stem Cell Isolation Technologies. TrAC Trends Anal. Chem..

[28] Song J., Song M., Kang T., Kim D., Lee L.P. (2014). Label-Free Density Difference Amplification-Based Cell Sorting. Biomicrofluidics.

[29] Wu M., Gao Y., Luan Q., Papautsky I., Chen X., Xu J. (2023). Three-dimensional Lab-on-a-foil Device for Dielectrophoretic Separation of Cancer Cells. Electrophoresis.

[30] Ozcelik A., Huang T.J. (2020). Acoustic tweezers for single-cell manipulation. Handbook of Single Cell Technologies.

[31] Akkoyun F., Gucluer S., Ozcelik A. (2021). Potential of the Acoustic Micromanipulation Technologies for Biomedical Research. Biomicrofluidics.

[32] Lee M.G., Shin J.H., Bae C.Y., Choi S., Park J.-K. (2013). Label-Free Cancer Cell Separation from Human Whole Blood Using Inertial Microfluidics at Low Shear Stress. Anal. Chem..

[33] Olofsson K., Hammarström B., Wiklund M. (2020). Acoustic Separation of Living and Dead Cells Using High Density Medium. Lab Chip.

[34] Yildizhan Y., Erdem N., Islam M., Martinez-Duarte R., Elitas M. (2017). Dielectrophoretic Separation of Live and Dead Monocytes Using 3D Carbon-Electrodes. Sensors.

[35] Zalis M.C., Reyes J.F., Augustsson P., Holmqvist S., Roybon L., Laurell T., Deierborg T. (2016). Label-Free Concentration of Viable Neurons, HESCs and Cancer Cells by Means of Acoustophoresis. Integr. Biol..

[36] Islam M., Brink H., Blanche S., DiPrete C., Bongiorno T., Stone N., Liu A., Philip A., Wang G., Lam W. (2017). Microfluidic Sorting of Cells by Viability Based on Differences in Cell Stiffness. Sci. Rep..

[37] Norouzi N., Bhakta H.C., Grover W.H. (2017). Sorting Cells by Their Density. PLoS ONE.

[38] Huh D., Bahng J.H., Ling Y., Wei H.-H., Kripfgans O.D., Fowlkes J.B., Grotberg J.B., Takayama S. (2007). Gravity-Driven Microfluidic Particle Sorting Device with Hydrodynamic Separation Amplification. Anal. Chem..

[39] Benincasa M.-A., Moore L.R., Williams P.S., Poptic E., Carpino F., Zborowski M. (2005). Cell Sorting by One Gravity SPLITT Fractionation. Anal. Chem..

[40] Herrmann N., Neubauer P., Birkholz M. (2019). Spiral Microfluidic Devices for Cell Separation and Sorting in Bioprocesses. Biomicrofluidics.

[41] Yoon Y., Lee J., Ra M., Gwon H., Lee S., Kim M.Y., Yoo K.-C., Sul O., Kim C.G., Kim W.-Y. (2019). Continuous Separation of Circulating Tumor Cells from Whole Blood Using a Slanted Weir Microfluidic Device. Cancers.

[42] Catarino S.O., Rodrigues R.O., Pinho D., Miranda J.M., Minas G., Lima R. (2019). Blood Cells Separation and Sorting Techniques of Passive Microfluidic Devices: From Fabrication to Applications. Micromachines.

[43] Gucluer S., Guler O. (2023). A Low-Cost Laser-Prototyped Microfluidic Device for Separating Cells and Bacteria. Appl. Sci..

[44] Javi F., Zaferani M., Lopez-Barbosa N., DeLisa M.P., Abbaspourrad A. (2022). Sheathless Inertial Microfluidic Cell Separation via a Serpentine–Contraction–Expansion Device Coupled with a Combinatorial Extraction Regulator. Microfluid. Nanofluidics.

[45] Akkoyun F., Ozcelik A. (2020). A Simple Approach for Controlling an Open-Source Syringe Pump. Eur. Mech. Sci..

[46] Reuß M., Josić D., Popović M., Bronn W.K. (1979). Viscosity of Yeast Suspensions. Eur. J. Appl. Microbiol. Biotechnol..

